# Experimental Infection of a North American Raptor, American Kestrel (*Falco sparverius*), with Highly Pathogenic Avian Influenza Virus (H5N1)

**DOI:** 10.1371/journal.pone.0007555

**Published:** 2009-10-22

**Authors:** Jeffrey S. Hall, Hon S. Ip, J. Christian Franson, Carol Meteyer, Sean Nashold, Joshua L. TeSlaa, John French, Patrick Redig, Christopher Brand

**Affiliations:** 1 USGS National Wildlife Health Center, Madison, Wisconsin, United States of America; 2 USGS Patuxent Wildlife Research Center, Laurel, Maryland, United States of America; 3 University of Minnesota School of Veterinary Medicine, Minneapolis, Minnesota, United States of America; University of Georgia, United States of America

## Abstract

Several species of wild raptors have been found in Eurasia infected with highly pathogenic avian influenza virus (HPAIV) subtype H5N1. Should HPAIV (H5N1) reach North America in migratory birds, species of raptors are at risk not only from environmental exposure, but also from consuming infected birds and carcasses. In this study we used American kestrels as a representative species of a North American raptor to examine the effects of HPAIV (H5N1) infection in terms of dose response, viral shedding, pathology, and survival. Our data showed that kestrels are highly susceptible to HPAIV (H5N1). All birds typically died or were euthanized due to severe neurologic disease within 4–5 days of inoculation and shed significant amounts of virus both orally and cloacally, regardless of dose administered. The most consistent microscopic lesions were necrosis in the brain and pancreas. This is the first experimental study of HPAIV infection in a North American raptor and highlights the potential risks to birds of prey if HPAIV (H5N1) is introduced into North America.

## Introduction

Waterfowl (Anseriformes), gulls, and shorebirds (Charadriiformes) are the primary reservoirs of low pathogenic avian influenza [1]. However, the role of wild birds in the ecology of highly pathogenic avian influenza virus (HPAIV) H5N1 is unclear. Many wild bird species have been found infected with HPAIV (H5N1), usually associated with a mortality event, but whether these species can act as reservoirs and if they have major roles in the long distance movement of the virus remains to be determined [2].

Wild waterfowl vary in their responses to infection with HPAIV (H5N1). Some species shed large amounts of virus, yet exhibit little to no obvious detrimental health effects [3]. On the other hand, highly susceptible species such as various species of swans (*Cygnus* spp.) and the wood duck (*Aix sponsa*), show 100% mortality within days of inoculation with HPAIV (H5N1) [4], [5].

One potential mechanism of HPAIV (H5N1) introduction into North America would be migratory waterfowl or shorebirds transporting the virus from Asia across the Bering Sea into Alaska, from whence it could be further disseminated throughout the continent by migratory birds. Species that prey on or scavenge sick or dying birds are also at risk of acquiring HPAIV (H5N1) from their food, as well as from the environment [6]. Raptors represent the apex of their food webs and thus, may be sensitive indicators of HPAIV (H5N1) activity. Not only may their broad geographic ranges make them susceptible to acquiring HPAIV (H5N1) but the migratory nature of many species may also contribute to long distance movement of HPAIV (H5N1). It is noteworthy that several species of raptors have been found either infected with, or dead from HPAIV (H5N1), including peregrine falcon (*Falco peregrinus*), Saker falcon (*Falco cherrug*), Hodgson's hawk eagle (*Spizaetus nipalensis*), common kestrel (*Falco tinnunculus*), buzzard (*Buteo buteo*), goshawk (*Accipiter gentilis*), and sparrowhawk (*Accipiter nisus*) [7]–[11]. To the best of our knowledge, the only controlled study examining the effects of HPAIV (H5N1) infection in raptor species was an experimental vaccination trial in captive gyr-saker falcon hybrids (*F. rusticolis x F. cherrug*) that showed that these birds were highly susceptible to HPAIV (H5N1) [10].

In addition, many raptor species are highly visible, endangered or threatened, and are politically and environmentally charismatic. Therefore it is important to experimentally assess the effects of HPAIV infection in raptor species to understand the potential roles they have in AI ecology and to assess risks to their populations. In this study, we experimentally inoculated American kestrels (*Falco sparverius*) with various doses of HPAIV (H5N1) and characterized infection in terms of survival, virus shedding, clinical signs, and pathology, with the goals of understanding the response of raptors to HPAIV infection and ultimately using American kestrels as a model species of HPAIV (H5N1) infection in falcons specifically, and raptors in general.

## Results

### Clinical signs and mortality

**Figure 1:**
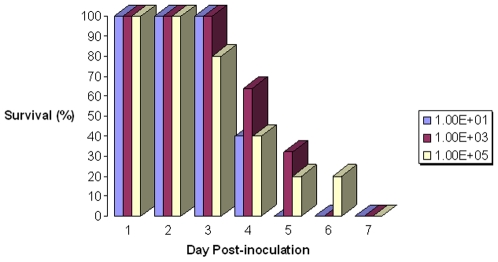
Survival of American kestrels infected with highly pathogenic avian influenza virus H5N1. Daily percentage of American kestrels surviving after infection with one of three doses of highly pathogenic avian influenza virus H5N1 (A/whooperswan/Mongolia/244/05).

All birds inoculated with virus died or showed severe clinical signs and were euthanized within 7 days of inoculation, regardless of dose administered (Figure 1). Clinical signs were similar in all inoculated subjects, consisting of feather fluffing, rhythmic side to side head movements, ataxia, head held at an angle, loss of appetite, loss of balance and motor control, and tremors. Birds also lost 8–10% of body mass in the 24 hours prior to death/euthanasia. On average, infected birds died or were euthanized for humane reasons between days 4–5 post-inoculation (DPI). There were no overt signs of any respiratory or intestinal disease such as dyspnea or diarrhea.

### Virus shedding

Within 24 hours after inoculation with HPAIV, all exposed birds were shedding virus orally (Table 1). Based on RT-PCR analysis, the amount of viral RNA shed orally was consistent, peaked on DPI 1, and gradually declined until death/euthanasia. RT-PCR analysis does not differentiate between viable, infectious virus and partial, inactivated or defective virus in the samples, therefore virus isolation from all swabs was performed. Virus isolation in embryonated egg culture showed that all oral swabs with measurable viral RNA had infectious virus present (Table 1). There was no statistical difference in shedding between doses (data not shown).

**Table 1:** Oral shedding of highly pathogenic avian influenza virus H5N1 by experimentally.

infected American kestrels.			
				Day Post-inoculation (DPI)				
Bird ID	Sex	Dose 1	DPI-0	DPI-1		DPI-2		DPI-3	DPI-4	DPI-5	DPI-6	DPI-7
2193	F	0	nc^2,3^	nc		nc		nc	nc	nc	nc	nc
2120	M	0	nc	nc		nc		nc	nc	nc	nc	nc
2192	F	1	nc	22.8*		21.0*		21.3*	24.0*			
2199	F	1	nc	24.8*		23.6*		22.5*	23.7*			
2177	M	1	nc	22.7*		23.4*		27.6*	27.9*			
2145	M	1	nc	20.1*		23.4		26.8*	27.0*	27.7*		
2160	F	1	nc	26.7*		24.5*		24.7*	26.8*			
2167	M	3	nc	21.1*		23.6*		24.1*	28.2*	23.1*		
2162	F	3	nc	21.7*		22.9*		24.7*	25.0*	27.2*	28.2*	
2130	M	3	nc	20.3*		25.7*		26.3*	27.8*	28.4*		
1994	F	3	nc	21.1*		23.4*		26.7*	24.4*	25.2*	27.9*	
2154	M	3	nc	19.7*		23.2*		25.0*	22.6*			
2123	M	3	nc	21.7*		23.1*		24.7*	25.4*			
2173	F	5	nc	20.5*		24.4*		22.7*				
2194	F	5	nc	19.7*		24.3*		26.6*	26.4*			
2189	M	5	nc	20.3 (3.0^4^)		25.4 (2.4)		23.5 (3.2)	22.8 (3.2)			
1992	M	5	nc	20.4*		22.3*		24.0*	25.9*	22.6*		
2127	M	5	nc	21.8*		27.8*		31.2*	28.5*	28.2*	30.5*	28.3*

1 Inoculation dose (log_10_ EID_50/_0.1 mL).

2 nc =  no Ct value obtained by H5 subtype specific RT-PCR analysis and confirmed in embryonated egg culture.

3 Virus amounts represented as RT-PCR Ct values.

4 Numbers in parentheses represent viral titers (log10 EID_50_/0.1 mL) as determined in embryonated egg culture.

*Presence of infectious virus confirmed by virus isolation in embryonated eggs.

Cloacal shedding had a different profile, typically lagging a day or more behind oral shedding before becoming detectable. The quantities of virus RNA were lower, rose more gradually, peaked on DPI 3–4, and declined more rapidly than oral shedding (Table 2). One kestrel (2127), inoculated with the high viral dose, shed similar amounts of viral RNA orally as other infected birds, yet shed virus cloacally on only DPI 3 and survived the longest of any subject inoculated (DPI 7). Many of the cloacal swabs, especially ones with lower amounts of viral RNA as measured by RT-PCR, did not have detectable infectious virus based on embryonated egg culture.

**Table 2:** Cloacal shedding of highly pathogenic avian influenza virus H5N1 by experimentally.

infected American kestrels.		
				Day Post-inoculation (DPI)			
Bird ID	Sex	Dose 1	DPI-0	DPI-1	DPI-2		DPI-3	DPI-4	DPI-5	DPI-6	DPI-7
2193	F	0	nc^2,3^	nc	nc		nc	nc	nc	nc	nc
2120	M	0	nc	nc	nc		nc	nc	nc	nc	nc
2192	F	1	nc	nc	33.2*		36.4	36.6			
2199	F	1	nc	nc	35.1*		37.0	30.5*			
2177	M	1	nc	nc	37.4		38.5*	38.8			
2145	M	1	nc	nc	33.0*		29.0	32.2*	35.8		
2160	F	1	nc	nc	31.9*		34.6*	33.1			
2167	M	3	nc	nc	24.5*		25.5*	21.9*	24.9*		
2162	F	3	nc	42.9	37.3*		nc	37.6	nc	nc	
2130	M	3	nc	nc	36.7*		38.3	38.4	nc		
1994	F	3	nc	39.4	37.7*		40.6	40.7	38.7	40.6	
2154	M	3	nc	41.8	32.9		33.5	28.9*			
2123	M	3	nc	nc	33.7*		35.1	33.9*			
2173	F	5	nc	34.8*	24.7*		23.8*				
2194	F	5	nc	nc	33.0*		37.8	39.1			
2189	M	5	nc	37.3	33.9* (1.4)^4^		37.8* (1.0)	38.4			
1992	M	5	nc	36.9	32.8*		34.3*	36.1	38.8		
2127	M	5	nc	nc	nc		40.1	nc	nc	nc	nc

1 Inoculation dose (log_10_ EID_50/_0.1 mL).

2 nc =  no Ct value obtained by H5 subtype specific RT-PCR analysis and confirmed in embryonated egg culture.

3 Virus amounts represented RT-PCR Ct values.

4 Numbers in parentheses represent viral titers (log10 EID50/0.1 mL) as determined in embryonated egg culture.

*Presence of infectious virus confirmed by virus isolation in embryonated eggs.

### Seroconversion

All birds were seronegative to avian influenza before inoculation. When possible, serum was collected from subjects immediately prior to euthanasia and analyzed by cELISA. Sera from all birds euthanized before DPI 4 were seronegative, and of those sera from birds that died on DPI 4, 3/5 (60%) had detectable antibodies to AI, independent of dose. All sera obtained from DPI 5 or afterwards were seropositive (data not shown). These data indicate that an immune response to HPAIV (H5N1), detectable by commercially available cELISA, occurs relatively quickly (by DPI 4–5) in infected birds. Because the kestrel colony where these birds were obtained has a history of West Nile virus (WNV) activity and infection with that virus could confound our analyses, we also screened the sera for WNV antibodies by PRNT_90_. One control kestrel (2193) was positive for WNV antibodies with a titer of 1∶320 and the rest of the birds were WNV seronegative. This non-inoculated control kestrel that was seropositive for WNV remained negative for HPAIV without any evidence of HPAIV (H5N1) shedding or seroconversion.

### Necropsy and Histopathology

Observable gross lesions were minimal, nonspecific and noted in only 4 kestrels. The respective lesions in these four separate kestrels were: 1) liver enlargement (approximately 1.25 times compared with control birds) 2) slight enlargement of spleen (5 mm diameter vs 3 mm in control birds); 3) congestion of the serosal surface of the duodenum and 4) of the upper jejunum. The control birds had noticeably greater amounts of mesenteric fat than all but one of the inoculated kestrels. All other tissues appeared overtly normal.

**Figure 2:**
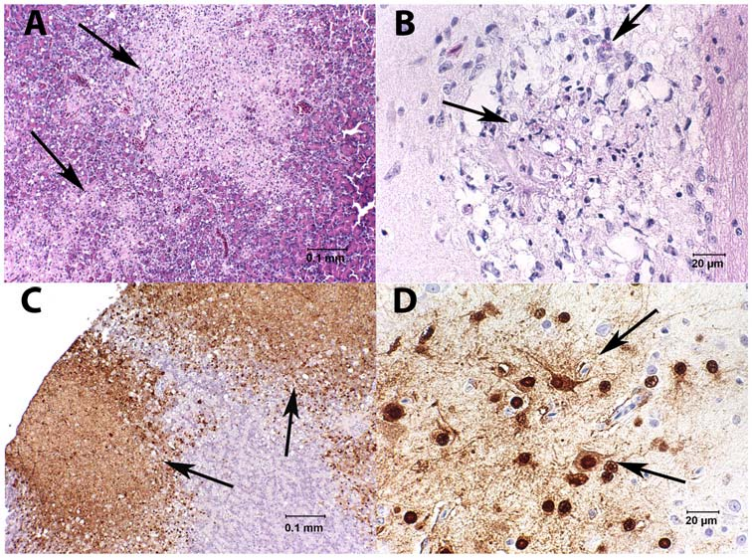
Tissues from American kestrels experimentally inoculated with highly pathogenic avian influenza virus H5N1. A. Pancreas with multifocal to coalescing areas of necrosis (arrows) (HE stain). B. Brain with multifocal cerebral necrosis (arrows) accompanied by a mild infiltration of heterophils (HE stain). C. Section of same pancreatic tissue as in Fig. 2A, stained by immunohistochemistry to identify influenza A virus antigen. Staining of influenza A antigen is intense in areas of necrosis (arrows). D. Immunohistochemical staining of influenza A virus antigen in brain tissue. Areas of brain necrosis are strongly positive (arrows).

All birds were examined histologically and a subset of tissues was stained for influenza A antigen by immunohistochemistry. Lesions observed in hematoxylin and eosin stained tissues were most consistent in the brain and pancreas. All influenza virus infected birds had meningitis and encephalitis ranging from mild, acute, multifocal necrosis to regionally severe necrosis associated with mild heterophilic inflammation (Fig. 2A). Lymphoplasmacytic perivascular cuffing was not a consistent finding but could be severe when present. The location of encephalitis was inconsistent between birds but taken together, was found in all regions of the brain. Microscopic lesions were seen in pancreatic sections from 12/16 HPAIV (H5N1) infected kestrels. In five of those, pancreatic lesions were mild and consisted of focal to multifocal apoptosis of acinar cells. The remaining 7 kestrels with pancreatic lesions had moderate to severe multifocal to coalescing pancreatic necrosis (Fig. 2B). Heterophils were not a predominant finding and when present were lightly scattered through the regions of necrosis. Nonspecific mild lymphoplasmacytic periportal inflammation and hepatocellular vacuolation were seen in 13 HPAIV (H5N1) infected kestrels and one kestrel that died on 3 DPI had multifocal acute hepatocellular necrosis accompanied by a mild infiltrate of heterophils. Mild vacuolation and widening of pulmonary interstitium was seen in 8 HPAIV (H5N1) infected kestrels. In addition, 2 of these had mild and one had moderate necrosis and heterophilic inflammation associated with airways. All other tissues showed only mild and inconsistent pathology, although every tissue type examined was affected in at least one or more birds. There were no lesions in the control kestrel that was seronegative for WNV. The kestrel that was seropositive for WNV had moderate multifocal lymphoplasmacytic encephalitis and mild meningitis, moderate lymphoplasmacytic myocarditis and interstitial nephritis. Necrosis of one sheathed artery in the spleen and non-specific mild periportal lymphoplasmacytic inflammation in the liver with light hemosiderin-like pigment accumulation in hepatocytes were also seen in the WNV seropositive kestrel.

Immunohistochemical staining was performed on tissues from 7 HPAIV (H5N1) infected kestrels. Tissues that had lesions identified in hematoxylin and eosin stained tissues stained positive for IHC influenza A antigen including brain and pancreas (Fig. 2C,D), lung, liver, and kidney. All cell types were positive in the regions of brain and lung involved. Acinar cells were the primary antigen-staining cells in the pancreas. The foci of necrotic hepatocytes stained positive for influenza A antigen in one liver and IHC staining was confined to Kupffer cells and serosal cells in another liver. The remaining 3/5 livers were negative. Influenza A virus antigen staining in both medullary and cortical cells of the adrenal gland was moderate in one of 5 birds and mild in another. Spleens were negative for IHC detectable viral antigen in 2/5 birds with only scattered positive cells in the spleens of the other 3. In addition, mild viral antigen staining was also present in, 2/3 trachea, 2/3 bone marrow, 4/6 heart. Although the outer muscular wall and serosa stained faintly with IHC in intestinal sections from 6/7 kestrels, antigen staining was seen in the mucosa of only one bird, and this was faint and inconsistent.

## Discussion

American kestrels were very susceptible to HPAIV (H5N1) infection. With every dose, 100% of inoculated birds died or had severe clinical signs and were euthanized within 7 days of inoculation. Infection was systemic, a finding confirmed by histological analyses of tissues. However, the most common observable signs were characteristic of neurological disease. Brain tissues had significant amounts of viral RNA and histology showed prominent inflammation and necrosis, consistent with encephalitis. All birds shed large amounts of virus orally and lesser amounts of virus cloacally. Considering the findings that oral shedding occurred earlier, was more consistent, and lasted longer than cloacal shedding, these data confirm that surveillance systems for HPAIV (H5N1) in raptors should include oral sampling, as has been recommended for other species [12].

Kestrels infected with HPAIV (H5N1) consistently exhibited clinical signs of central nervous system disease which has not been previously reported in HPAIV (H5N1) infected falcons. Additionally, in our study, a range of biologically relevant doses of virus was examined. Because all inoculated birds died, regardless of dose, the minimum infectious and lethal doses could not be calculated, but was less than 10 EID_50_, the smallest virus dose administered. This level of exposure could readily be encountered by wild birds through consumption of infected prey, contact with infected conspecifics, or by contact with a HPAIV (H5N1) contaminated environment.

Should HPAIV (H5N1) reach North America, the risk to kestrel populations is probably relatively minor given their feeding and social behavior, the large geographic range, and relatively large population size of this species. However, local kestrel populations in the United States are declining for unknown reasons and may have been negatively impacted by the introduction and spread of West Nile virus [13]. The introduction of HPAIV (H5N1) could have an additional negative impact on this species' status.

Other species of raptors such as eagles, peregrine falcons, accipiters, hawks, vultures, and California condors (*Gymnogyps californianus*), that prey on or scavenge waterbirds, and whose populations are much smaller and confined to more localized geographic areas, would have greater risk of HPAIV (H5N1) adversely impacting their populations. Raptors are the apex of their food webs, effectively sampling sick, dying, or dead prey and, thus, represent a natural surveillance system that likely targets those subjects most likely to have had HPAIV exposure. Traditional live-bird or hunter-killed waterfowl influenza surveillance systems overwhelmingly sample healthy birds. Monitoring raptor species, especially raptor mortality, may be an additional method of surveillance for HPAIV (H5N1). Clearly, understanding how HPAI would impact raptor species and the roles of these species in the disease ecology of influenza is important and warrants additional research. A captive breeding colony and the corresponding ease of housing and handling make American kestrels accessible and available in sufficient numbers for experimental and/or vaccine studies without the expense of taking birds from the wild. Thus, American kestrels may represent a suitable model species for HPAIV (H5N1) infection studies in falcons specifically and raptors in general.

## Materials and Methods

### Subjects

Immature American kestrels (4–5 months) were acquired from a captive breeding colony at the USGS Patuxent Wildlife Research Center, Laurel, MD and transported to the USGS National Wildlife Health Center, Madison, WI. Birds were randomly assigned to individual isolator cages (1/cage) in a Biosafety level 3+ facility and allowed to acclimate for 7 days with water and food provided *ad libitum*. Birds were fed Nebraska Brand Bird of Prey Diet (Central Nebraska Packing, Inc., North Platte, NE) supplemented with a dead mouse every other day. All procedures, housing, transport, and care were approved by the institutional animal care and use committee.

### Highly pathogenic avian influenza virus isolate and virus inoculation

Kestrels were inoculated intranasally and intrachoanally with one of three doses of HPAIV (H5N1) (A/whooperswan/Mongolia/244/05) in a total volume of 100 µl. Two uninoculated birds served as controls. Inocula were diluted in brain heart infusion broth with penicillin/streptomycin and viral titers confirmed by the method of Reed and Muench [14] in embryonated chicken eggs [15]: 10^1.0^ EID_50_/100 µl (5 birds), 10^3.0^ EID_50_/100 µl (6 birds), 10^5.0^ EID_50_/100 µl (5 birds).

### Sampling

All birds were weighed daily and monitored as needed to ascertain health status. Birds exhibiting severe disease signs, especially neurological signs, were euthanized for humane reasons. Serum samples were collected from all birds on day post-inoculation (DPI) 0 and, when possible, immediately prior to euthanasia by CO_2_ asphyxiation. Sera were stored at -20°C. Daily cloacal and oropharyngeal swabs were taken using Dacron tipped applicators, placed in cryovials containing viral transport media (Hanks Balanced Salt Solution, 0.05% gelatin, 5% glycerin, 1500 U/mL penicillin, 1500 µg/mL streptomycin, 0.1 mg/mL gentamicin, 1 µg/mL fungizone), and stored at −80°C until analyses.

### Serology

The pre-inoculation (day 0) and final serological status were determined using the IDEXX Multi-Species ELISA kit according to the manufacturer's directions (IDEXX Laboratories, Westbrook, ME). Plaque reduction neutralization assays to detect West Nile virus antibodies were performed according to the procedures outlined in [16].

### RNA extraction and real time reverse transcription polymerase chain reaction (RT-PCR)

Viral RNA was extracted from cloacal and oropharyngeal swabs using the MagMAX^TM^-96 AI/ND Viral RNA Isolation Kit (Ambion, Austin, TX) following the manufacturer's procedures. Real time RT-PCR was performed using the published procedures, primers, and probe of Spackman et al. [17] for the detection of HPAIV (H5N1). RT-PCR assays used reagents provided in the Qiagen OneStep^®^ RT-PCR kit (Qiagen, Valencia, CA) and were performed on a Stratagene Mx3005P thermal cycler.

### Necropsy, immunohistochemical, and histological analyses

All 16 inoculated kestrels and the two uninoculated controls were examined at necropsy when, using sterile procedures, portions of brain, trachea, lung, heart, liver, kidney, adrenal, spleen, duodenum, pancreas, jejunum, cecal tonsil, and cloaca with bursa of Fabricius were collected for histopathology. Tissues were fixed in 10% neutral buffered formalin, embedded in paraffin, sectioned at 5 µm, stained with hematoxylin and eosin, and examined by light microscopy. Additional portions of brain, intestine, liver, lung, spleen, and kidney were collected for RNA extraction and RT-PCR analyses as described above.

Immunohistochemistry was processed at the Histology Laboratory, Department of Pathology, College of Veterinary Medicine, University of Georgia. Following deparaffinization, proteinase K was used for antigen retrieval and endogenous peroxidase was blocked using 3% hydrogen peroxide (H312-500, Fisher Scientific, Fair Lawn, NJ). Mouse monoclonal antibody to Influenza A virus nucleoprotein (C65331M, Biodesign International, Saco, ME 1 mg/ml), diluted 1∶200 using Dako® Antibody Diluent (S0809, Dako, Carpinteria, CA), was applied to slides for 60 minutes. This was followed by biotinylated horse anti-mouse IgG (BA-2001, Vector Labs, Burlingame, CA) and streptavidin conjugated to horseradish peroxidase (Dako's LSAB® 2; K1016, Dako, Carpinteria, CA). The substrate-chromogen system used was DAB (K3466, Dako, Carpinteria, CA) and slides were counterstained with Gills II hematoxylin. Positive tissue controls consisted of formalin fixed, paraffin-embedded heart from avian influenza-infected chicken. As a negative control, primary antibody was substituted with Universal Negative Control (N1698, Dako, Carpinteria, CA).
